# Hourglass Bladder Deformity Induced by Chronic Ketamine Abuse

**DOI:** 10.5334/jbsr.3595

**Published:** 2024-05-16

**Authors:** Kevin Badran, Olivier Rahier, Emmanuel Agneessens

**Affiliations:** 1 Radiology department, Cliniques universitaires Saint-Luc, Av. Hippocrate 10, 1200 Bruxelles, Belgique; 2 Urology department, Clinique Saint-Pierre Ottignies, Av. Reine Fabiola 9, 1340 Ottignies-Louvain-la-Neuve, Belgique; 3 Radiology department, Clinique Saint-Pierre Ottignies, Av. Reine Fabiola 9, 1340 Ottignies-Louvain-la-Neuve, Belgique

**Keywords:** Bladder, ketamine, hourglass, CT scan, ultrasound

## Abstract

Regular and chronic use of ketamine causes inflammatory changes in the urinary tract. Imaging has a crucial role in the assessment of this pathology. A particular imaging characteristic of an hourglass-shaped bladder is highlighted in three cases of ketamine intoxication.

*Teaching point:* Three cases of ketamine intoxication with a characteristic bladder deformation are reported.

## Introduction

Ketamine, a non-competitive *N*-methyl-D-aspartic acid receptor antagonist, was discovered in 1961 and is used in medical practice as a clinical anaesthetic [1]. However, it can be used in an illicit manner for recreational purposes, particularly for its psychodysleptic effects [2]. According to the ‘Global Drug Survey 2022 7 Year Drug Trend Report’, an increase in ketamine consumption was observed between 2015 and 2022. This article presents three cases of ketamine intoxication responsible for a characteristic an hourglass-like deformity of the bladder.

## Case Report

A 23-year-old female patient consulted for loss of bladder compliance with urinary urgency, haematuria and urinary frequency for several months. She mentioned a decreasing use of chronic ketamine (for recreational use): a dose of less than 1 g per week currently compared to 1 to 2 g per day previously (2016–2020), associated with occasional consumption of ecstasy and LSD (lysergic acid diethylamide).

**Figure 1:**
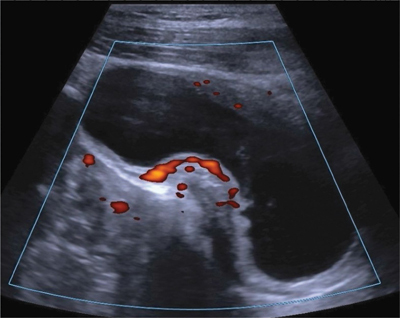
A 23-year-old patient. Color Doppler ultrasound in the sagittal section showing the ‘hourglass’ appearance due to the localised hypervascular bladder wall thickening.

An ultrasound of the urinary tract (Figure 1) showed a thickening of the bladder wall predominantly in its middle portion, an ‘hourglass’ appearance, and hyperaemia on a Color Doppler. Bilateral hydroureteronephrosis was also present.

The second case, a 28-year-old male patient, consulted for dysuria, haematuria and occasional mictalgia. He used ketamine chronically (5–10 g per day for 5 years) and occasionally cocaine.

**Figure 2:**
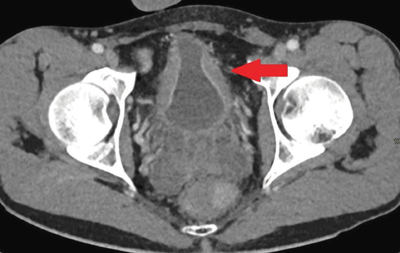
A 28-year-old patient. A contrast-enhanced abdominal CT scan showing the hypervascular mucosa and wall thickening of the middle part of the bladder with an ‘hourglass’ appearance (red arrow).

A contrast-enhanced abdominal computed tomography (CT) scan illustrated slight symmetrical wall thickening and an hourglass shape (Figure 2).

The third case, a 26-year-old male patient, presented to the emergency care with suprapubic pain for 3 days. The patient had been using ketamine for years (exact duration and quantity unknown).

**Figure 3:**
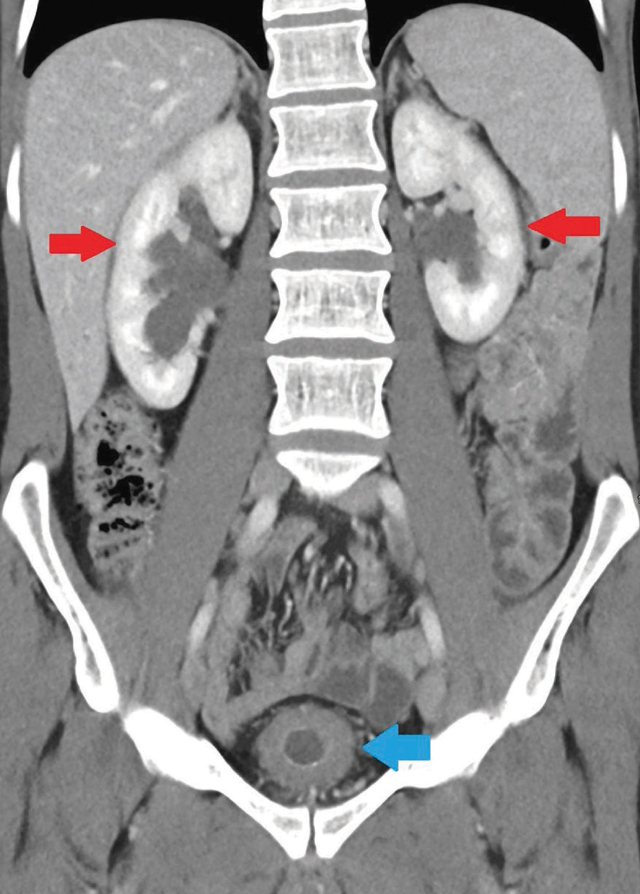
A 26-year-old patient. Contrast-enhanced CT showing the shrunken bladder with thickened wall (blue arrow) associated with bilateral hydronephrosis (red arrows).

A contrast-enhanced abdominal CT (Figure 3) showed mucosal hyperaemia and thickening of the bladder wall in the middle part. There was also bilateral hydronephrosis with dilated ureters up to the vesical junction.

## Discussion

Regular and chronic use of ketamine provokes inflammatory changes in the bladder wall causing voiding problems. Urinary symptoms include dysuria, urinary frequency, urgency, pelvic pain and haematuria [3].

Imaging has a crucial role in the assessment of urinary tract involvement.

Ultrasonography may show (localised) bladder wall thickening, calcifications and hydronephrosis.

A CT of the urinary tract is essential for the evaluation of haematuria and the extent of involvement of the urinary tract. The most observed signs in the lower urinary tract include enhancement of the bladder mucosa, diffuse or localised thickening of the bladder wall, decreased bladder volume, perivesical inflammation and even a vesicovaginal fistula. The involvement of the upper urinary tract includes hydronephrosis (without obstructive calculi) and thickening of the ureteral wall. Chronic hydronephrosis may be extremely harmful, and lead to irreversible renal failure [4].

The diagnosis of ketamine-induced uropathy is challenging because its symptoms can mimic other conditions such as urinary tract infection, ulcerative or interstitial cystitis, overactive bladder syndrome or ‘Bladder Pain Syndrome’.

Cessation of the ketamine intake is the first and fundamental step in treatment [5]. In selected cases, dedicated surgery may be necessary to support relieve symptoms [6].

## Conclusion

Imaging has a crucial role in the evaluation of urinary tract damage in patients with uropathy induced by chronic ketamine consumption. Three cases of ketamine intoxication responsible for a characteristic hourglass deformity of the bladder are reported.
